# Potent Induction of Arabidopsis thaliana Flowering by Elevated Growth Temperature

**DOI:** 10.1371/journal.pgen.0020106

**Published:** 2006-07-07

**Authors:** Sureshkumar Balasubramanian, Sridevi Sureshkumar, Janne Lempe, Detlef Weigel

**Affiliations:** 1 Department of Molecular Biology, Max Planck Institute for Developmental Biology, Tübingen, Germany; 2 Plant Biology Laboratory, Salk Institute for Biological Studies, La Jolla, California, United States of America; University of Southern California, United States of America

## Abstract

The transition to flowering is an important event in the plant life cycle and is modulated by several environmental factors including photoperiod, light quality, vernalization, and growth temperature, as well as biotic and abiotic stresses. In contrast to light and vernalization, little is known about the pathways that mediate the responses to other environmental variables. A mild increase in growth temperature, from 23 °C to 27 °C, is equally efficient in inducing flowering of *Arabidopsis* plants grown in 8-h short days as is transfer to 16-h long days. There is extensive natural variation in this response, and we identify strains with contrasting thermal reaction norms. Exploiting this natural variation, we show that *FLOWERING LOCUS C* potently suppresses thermal induction, and that the closely related floral repressor *FLOWERING LOCUS M* is a major-effect quantitative trait locus modulating thermosensitivity. Thermal induction does not require the photoperiod effector *CONSTANS,* acts upstream of the floral integrator *FLOWERING LOCUS T,* and depends on the hormone gibberellin. Analysis of mutants defective in salicylic acid biosynthesis suggests that thermal induction is independent of previously identified stress-signaling pathways. Microarray analyses confirm that the genomic responses to floral induction by photoperiod and temperature differ. Furthermore, we report that gene products that participate in RNA splicing are specifically affected by thermal induction. Above a critical threshold, even small changes in temperature can act as cues for the induction of flowering. This response has a genetic basis that is distinct from the known genetic pathways of floral transition, and appears to correlate with changes in RNA processing.

## Synopsis

When to flower is an important decision in the life cycle of a plant, as it determines the plant's reproductive success. Not surprisingly, plants closely monitor the state of their life cycle along with the external environment in order to determine the onset of flowering. Several factors including light, temperature, and abiotic stress are known to affect the timing of flowering. The authors show that growth temperatures above a finely tuned threshold can rapidly trigger flowering, bypassing the need for other inductive stimuli such as day length. Exploiting a combination of Mendelian genetics, natural variation, and genomics, they show thermal induction of flowering to have a unique genetic basis. Genomic responses to temperature and light during floral induction differ, and temperature-specific changes include alterations in RNA processing.

## Introduction

The postembryonic developmental program of plants is extraordinarily flexible and can change dramatically in response to many environmental factors. Examples of such variables are light quantity (day length), light quality (red–far-red ratio), vernalization (exposure to winter temperatures for several weeks) and ambient growth temperature [1,2]. In addition, flowering is also affected by nitrous oxide and various stresses, including biotic (e.g., pathogens) and abiotic stress [3–5]. Extensive genetic analysis of laboratory-induced mutations as well as naturally occurring genetic variants has identified at least four distinct pathways controlling flowering in the reference plant Arabidopsis thaliana [6]*.* The photoperiodic pathway receives inputs from the circadian clock and day length, and the nuclear protein CONSTANS (CO) integrates its effects. Vernalization promotes flowering by enabling stable repression of *FLOWERING LOCUS C (FLC),* a potent suppressor of flowering. In winter annual accessions of *Arabidopsis, FLC* levels are high due to activation by *FRIGIDA (FRI).* Loss of function of *FRI* or attenuation of *FLC* contributes to a rapid-cycling behavior in many *Arabidopsis* accessions, including the commonly used laboratory strains Landsberg *erecta* and Columbia [7–12]. A series of autonomous pathway genes promote flowering in a photoperiod-independent manner, also via suppression of *FLC* levels. Finally, the hormone gibberellin (GA) is essential in a fourth pathway, which controls flowering redundantly with the photoperiod pathway. All of these pathways appear to converge on a small number of integrators, the flowering-time genes *FLOWERING LOCUS T (FT)* and *SUPPRESSOR OF OVEREXPRESSION OF CONSTANS 1 (SOC1),* and the floral meristem identity gene *LEAFY* [1,13].

In contrast to light- and vernalization-dependent flowering, less is known about how other factors modulate the transition to flowering [14]. Some progress has been made in understanding how moderate changes in growth temperature affect flowering*.* Plants with a defect in the red-light receptor phytochrome B (PHYB) flower early at 23 °C, but not at 16 °C [15]. In contrast, higher temperatures ameliorate the late-flowering phenotype of plants that lack the blue light receptor *CRYPTOCHROME 2 (CRY2),* apparently because the redundantly acting far-red-light receptor *PHYTOCHROME A (PHYA)* is not able to promote flowering at lower temperatures [16]. Such a flowering behavior is found in many wild accessions, which cluster in their response to different environments with *cry2* mutants [11]. The differential flowering phenotype of *phyB* and *cry2* mutants contrasts with the temperature-insensitive late flowering of autonomous pathway mutants [16].

Here, we show that a modest increase of 2 °C to 4 °C in ambient growth temperature beyond the common laboratory condition of 23 °C potently triggers flowering in the absence of photoperiodic cues. There is extensive natural variation in this response, and we identify loci that contribute to this response in wild accessions of *Arabidopsis thaliana.* We demonstrate that the floral repressors *FLC* and *FLM* differ in their effects on thermal induction, and that the thermal response is integrated downstream of *CO* at *FT.* Microarray analyses confirm that the genomic responses to photoperiodic and thermal induction differ, and identify unique sets of genes that are activated or repressed in response to either cue.

## Results/Discussion

### Mild Temperature Increase beyond 23 °C Potently Induces Flowering in Short Days

**Figure 1:**
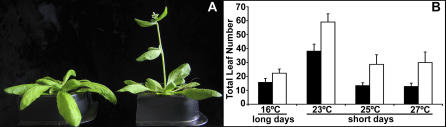
Flowering Response of L*er* and Col under Different Temperature Regimens (A) Arabidopsis thaliana strain L*er* grown in 23 °C short days (left) and 27 °C short days (right). (B) Flowering time of L*er* (black bars) and Col (white bars) in different conditions. Error bars indicate standard deviation.

**Figure 2:**
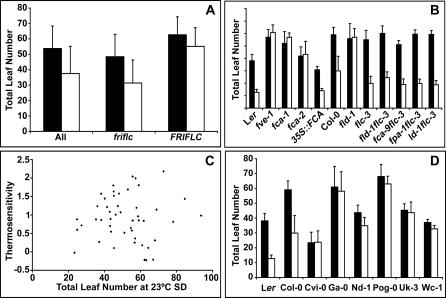
Natural Variation in Thermal Response (A) Mean flowering time of accessions in short days at 23 °C (black bars) and 27 °C (white bars). Error bars indicate standard deviation. All, all strains; *friflc,* subset of strains that have nonfunctional alleles at *FRI* and/or *FLC; FRIFLC,* subset of strains with putatively functional alleles at *FRI* and *FLC*. Student's *t* test shows the difference between 23 °C and 27 °C to be significant for the first two groups (*p* < 0.0001), but not for the last. (B) Flowering times of single and double mutants of the autonomous pathway and *flc-3* at 23 °C (black bars) and 27 °C (white bars). *fpa-*T refers to a T-DNA allele of *fpa* in the Col background. Genotypes are grouped based on their genetic background, with L*er* and Col controls shown to the left of each group. (C) Natural variation in the thermal sensitivity of accessions. Thermosensitivity is plotted as a function of TLN in short days at 23 °C. (D) Flowering times of temperature-insensitive accessions among strains that lack functional *FRI/FLC.*

Arabidopsis thaliana is commonly cultivated in the laboratory at temperatures of 20 °C to 23 °C, even though its natural distribution is largely in areas that have a lower mean temperature [17]. While growing wild-type Landsberg *erecta* (L*er*) and Columbia (Col) plants at a range of temperatures in short days, we noticed that a modest increase in temperature from 23 °C to either 25 °C or 27 °C had a dramatic effect on flowering (Figure 1A). As reported before, the two common laboratory strains L*er* and Col flowered much later in 23 °C 8-h short days than 16 °C 16-h long days [11]. L*er* flowered at about the same time in 25 °C or 27 °C short days as in 16 °C long days, while Col flowered only slightly earlier in 16 °C long days compared to 25 °C and 27 °C short days (Figure 1B). The similar behavior is seen both when measuring days to flowering (chronological time) as well as total leaf number produced before the first flower on the main stem (physiological age), indicating that this is not simply due to differences in growth rate (unpublished data). Since it is known that flowering-time mutants and wild strains of *Arabidopsis* vary in their long-day flowering behavior under different temperature regimens [11,16], we assessed the thermal response of about 50 mutants and 52 wild accessions in short days. As with many other environmental responses, there is extensive variation in thermal response (Table S1). However, strains flowered on average earlier at 27 °C compared to 23 °C short days, and there was a significant difference in mean total leaf number between 23 °C and 27 °C (Figure 2A). This difference was also seen when total leaf number was partitioned into rosette and cauline leaf numbers.

### Effects of *FRI/FLC* on Thermal Induction of Flowering

*FLC* is a potent repressor of flowering. Together with its upstream regulator *FRI, FLC* plays an important role in natural variation of Arabidopsis thaliana flowering [7–9,11,12,18–21]. Since the effect of *FRI/FLC* is reduced, but still significant in short days, we first assessed whether strains that differ in *FRI/FLC* status vary in thermal response. We found a significant difference in the mean total leaf number at 23 °C versus 27 °C only among the strains that have lesions at *FRI* or *FLC* (Figure 2A). Since *flc-*3 knockout mutants can still respond to thermal induction in a manner similar to the parental Col line, thermal induction cannot be simply mediated by suppression of *FLC.* We therefore asked whether elevated *FLC* levels could attenuate thermal induction. A well-known way to increase *FLC* levels even in the absence of functional *FRI* is through mutations in the autonomous pathway [1]. Mutants with defects in this pathway did not respond to thermal induction of flowering (Figure 2B). If higher *FLC* levels are indeed responsible for the failure of autonomous pathway mutants to respond to elevated temperatures, a mutation in *FLC* should suppress their nonresponsiveness to thermal induction. Indeed, double mutants of *flc*-3 with autonomous pathway mutants in the Col background showed a response similar to that of *flc-*3 single mutants, flowering substantially earlier in 27 °C than 23 °C short days (Figure 2B). In addition, plants that overexpress *FCA,* an autonomous pathway gene, responded well to thermal induction (Figure 2B).

It has recently been demonstrated that *FLC* mediates natural variation in temperature compensation of the circadian clock [22]. *FLC* lengthens the period of the clock at higher temperatures, which should lead to a delay in flowering, consistent with our observations. If *FLC* indeed modulates sensitivity to temperature, one might expect that the mean sensitivity to temperature will vary between the lines with functional or nonfunctional alleles at *FRI/FLC.* We found this to be the case, with the lines that have lesions at *FRI*/*FLC* being more sensitive than the lines with putatively functional *FRI*/*FLC* (ANOVA, *p* = 0.02). Taken together, these results suggest a role of *FLC* in suppressing thermal induction. In addition, these results indicate that the failure of autonomous pathway mutants to respond to thermal induction appears to be largely due to their elevated *FLC* levels.

We next asked in several ways whether accessions differ in their response to temperature. The reaction norms and the thermal sensitivities of accessions indicated that there is considerable variation in their response to thermal induction (Figure 2C and Table S2). We then ranked the accessions based on their flowering time at 23 °C and 27 °C. While there is a general correlation between flowering times in the two conditions, some strains ranked very differently in 23 °C versus 27 °C (Table S2). These findings confirm that there is a significant genotype × environment (G × E) interaction in thermal response. Finally, by comparing flowering times at 23 °C and 27 °C, we identified strains that do not respond to thermal treatment.

Since *FLC* is a potent suppressor of thermal induction, we looked for nonresponsive accessions among those that carry nonfunctional alleles at either *FRI* or *FLC* (Figure 2D). Of six unresponsive strains, Uk-3, Pog-0, and Cvi-0 have high *FLC* levels in spite of an *FRI* deletion [11]. Because the higher *FLC* levels likely explain the thermal insensitivity of these three accessions, we focused our further analysis on other temperature non-responsive strains.

### *FLM* Modulates Thermal Sensitivity

**Figure 3:**
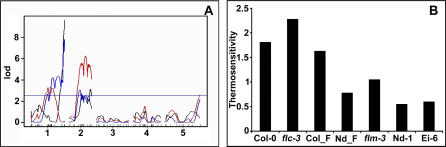
Effect of *FLM* on Thermal Sensitivity in Short Days (A) QTL maps of NdC RILs for TLN in 27 °C short days (red lines) and 23 °C short days (black lines) and for thermal sensitivity, as expressed by the slope of the regression line mean over the environmental mean in arbitrary units (blue lines). The phenotype data for the 23 °C map are from [24]. The prominent QTL corresponding to *FLM* on Chromosome 1 disappears at 27 °C, while the QTL on Chromosome 2 becomes more significant. The QTL for thermal sensitivity colocalize with *FLM.* A likelihood of odds threshold determined after 1,000 permutations is given. The same threshold was obtained for each of the phenotypes. (B) Thermal sensitivity of various genotypes as above. Col_F and Nd_F refers to the mean sensitivity of NdC recombinant inbred lines that are homozygous for the Col wild-type allele (Col_F) and homozygous for the Nd-1 *FLM* deletion (Nd_F). For comparison the sensitivity of *flc-3* is shown. *flm-3* is a T-DNA insertion allele at *FLM* locus in Col background. The last genotype is the accession Ei-6, which has the same *FLM* deletion as Nd-1. The effect of loss of *FLM* in different backgrounds varies considerably between backgrounds, indicating natural variation in this pathway.

Since recombinant inbred lines were available for Col-0 crossed to Nd-1, a temperature-insensitive strain with low *FLC* levels, we decided to perform quantitative trait locus (QTL) mapping using Niederzenz-1/Col (NdC) recombinant inbred lines (RILs) [23]. QTL mapping experiments have led to the identification of a deletion of the floral repressor *FLOWERING LOCUS M (FLM)/MADS AFFECTING FLOWERING1 (MAF1)* as a major cause for early flowering of Nd-1 in short days at 23 °C [24]. Since *FLM* is similar in sequence to *FLC,* which represses thermal induction, we expected that *FLM* would also inhibit temperature responsiveness. Contrary to our expectations, the effect of *FLM,* the major effect QTL in 23 °C short days, was masked in 27 °C short days, and the QTL was no longer detectable (Figure 3A). Consistently, there is no significant difference in the mean flowering time of plants with or without the *FLM* deletion at 27 °C (not shown), indicating that *FLM* modulates the sensitivity to temperature. To confirm this assumption, we calculated the thermal sensitivities of each of the RILs and asked whether a QTL for thermal response colocalizes with the *FLM* locus, which was indeed the case (Figure 3A). In contrast to *FLC,* where strains with low expression levels respond more strongly to thermal induction, lines without *FLM* respond less well to temperature compared to *FLM* wild-type strains (Figure 3B). This finding suggests that temperature acts in the same genetic cascade as *FLM.* Temperature might suppress the repressive effect of *FLM* on flowering. Alternatively, temperature might act downstream of *FLM* to bypass the repressive effect of *FLM.*

The response to temperature is reduced, but not eliminated in the NdC lines with the *FLM* deletion, compared to lines with the wild-type allele, indicating that other factors contribute to thermal response as well (Figure 3B). We identified Ei-6, another strain that flowers early in short days, as having the same deletion as Nd-1 (not shown). Ei-6 also has a reduced thermal response (Figure 3B). However, Ei-6 flowers even faster than Nd-1 in 23 °C short days. Our earlier analysis of F2 populations derived from a cross between Ei-6 and Col had pointed to a complex genetic basis of the early flowering behavior of Ei-6 [11], consistent with the hypotheses that there are natural modifiers for *FLM* effects.

### Thermal Induction Does Not Depend on *CO*

**Figure 4:**
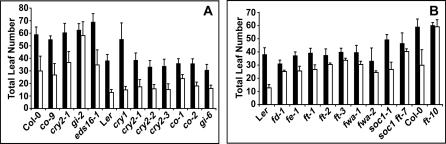
Effect of Different Genetic Pathways on Flowering Time in 27 °C Short Days Flowering time of mutants with defects in flowering time genes in 23 °C short days (black bars) and 27 °C short days (white bars). L*er* and Col controls are included both panels. (A) Mutants with defects in the photoperiod pathway, and *eds16–1. co-1* is in a mixed background of Col-0 and L*er*. (B) Mutants with defects in floral integrators. *ft-2* and *ft-7* are two independently isolated alleles with the same mutation.

*FLM* functions independently of the autonomous and vernalization pathways, but interacts with the photoperiod pathway [25]. Therefore, we tested the effects of photoperiod mutants, *gigantea (gi), co, cry1,* and *cry2* [26]. *GI* encodes a nuclear protein with several roles in light-mediated and stress responses [27–30], while CO functions as the principal output of the photoperiod pathway by integrating circadian with light information [31,32]. The CRY2 photoreceptor functions primarily in flowering control, while CRY1 controls both seedling and flowering responses to blue light [33,34]. A strong thermal response is retained in plants that have mutations in the CRY1 or CRY2 photoreceptors (Figure 4A). Like the *cry* mutants, *phyA, phyB*, and *phyC* mutations do not interfere with the thermal response (unpublished data). *co* mutants in either the Col or L*er* background flower considerably earlier in 27 °C compared to 23 °C short days (Figure 4A). Similarly, the *gi-6* allele induced in L*er* shows a thermal response, although this is not the case for the *gi-2* allele induced in Col. To determine whether this difference is due to a direct role of *GI* in thermal response or due to an allele specific effect of *gi-2*, we tested another strong allele, *gi-3*, in the L*er* background. *gi-3* mutants flowered earlier and similar to *gi-6* at 27 °C (Table S1). Thus, the absence of a thermal response in *gi-2* plants could be due to natural variation between L*er* and Col with respect to *GI* function, or caused by an allele specific effect of *gi-2*, rather than indicating a direct role for *GI* in thermal response. An allele-specific effect is consistent with the recent observation that *gi-2* differs from other *gi* alleles in its effects on the circadian clock [35]. However, we cannot rule out the possibility that the response of the L*er* alleles is due to remaining partial *GI* activity in *gi-3* or *gi-6*.

### Thermal Induction Is Mediated by *FT*

Flowering pathways converge at the level of the so-called integrators. Among these, *FT* and *SOC1* have crucial roles in flowering time, while *LEAFY* functions primarily in floral identity [36–40]*.* In addition to *ft* and *soc1* mutants, we assayed *fwa* mutants, which have a reduced response to *FT* activity [37,38], along with plants with a mutation in *FD,* which mediates *FT* activity [21,41], as well as *fe* mutants, which have a similar genetic behavior as *ft* [42]. All these mutants showed a reduced response to 27 °C short days (Figure 4B), suggesting that thermal induction, like the other known floral induction pathways, acts upstream of the integrators.

*soc1–1 ft*-*7* double mutants flowered in 27 °C short days at a similar time as wild-type did in 23°C short days, but much later than wild-type in 27 °C short days (Figure 4B). It has recently been suggested that the available *ft* alleles in the L*er* background are hypomorphic alleles, and that the additive effect of *soc1–1 ft-7* double mutants is simply due to *ft-7* not being a null allele [43]. Consistent with this hypothesis we found that the RNA null allele *ft-10* in the Col background is completely insensitive to temperature (Figure 4B), indicating that thermal induction is mediated primarily by *FT.*

All *co* alleles tested, *co-2* and *co-8* in L*er*, and *co-9* in Col, have a pronounced response to temperature, indicating that thermal induction is clearly independent of *CO.* Thus, temperature must affect *FT* in at least in two ways. First, temperature modulates the effects of floral repressors such as *FLC.* Second, temperature leads to a photoperiod independent activation of *FT*. Consistent with this hypothesis, we find mRNA levels of *FT* to be more than ten-fold at 27 °C compared to 23 °C short days (Figure S1). Circadian oscillation of *FT* was not affected; its relative levels were higher at all time points. The increase in *FT* expression levels could be at least partially responsible for the thermal induction of flowering, consistent with the effects of an *ft* mutation on this process.

### GA and Thermal Induction

GA acts redundantly with the photoperiod pathway in promoting flowering, and they are especially important in short days [40,44,45]. Plants with a dominant mutation in the GA response factor *GA INSENSITIVE (GAI),* which have a reduced response to GA, flower much later than wild-type under normal short day conditions [44]. Short-day flowering of *gai-D* plants was still accelerated by 27 °C (Table S1). GA-deficient *ga1–3* mutants, which cannot flower at all in regular short days [44], also did not respond to thermal induction. Thus, thermal induction cannot overcome the requirement for GA in short days. Given that the *ga1–3* phenotype can be suppressed by overexpression of *FT* [40], this observation suggests either that thermal induction of *FT* is not sufficiently strong to bypass the GA requirement, or that thermal induction does not act exclusively through *FT,* at least in the L*er* background. Consistent with this, a small thermal response was retained by *ft* mutants in L*er* background (Figure 4B).

### Thermal Induction Is Independent of SA-Mediated Stress Signaling

Because temperatures in the native range of *Arabidopsis* are on average well below 25 °C [17], we were curious whether flowering upon thermal induction could be due to a stress response. Many stress responses in plants are promoted by the hormone abscisic acid (ABA) [46]. ABA has recently been shown to act through the *FLC* regulator FCA [47]. ABA, however, represses flowering, which is inconsistent with a positive role of ABA in accelerating flowering in response to higher temperatures.

Another stress hormone that has recently been implicated in flowering control is salicylic acid (SA), which is required for activation of flowering by UV-C light stress. The effects of SA signaling on known flowering regulators are complex [4]. To determine whether thermal induction of flowering might be caused by the SA pathway, we tested the thermal response of mutants in which SA-dependent responses are blocked. Mutations in *ENHANCED DISEASE SUSCEPTIBILITY1 (EDS1)* were initially isolated because they are required for certain types of pathogen resistance [48]. More recently, *EDS1* has been shown to play important roles in responses to several abiotic stresses as well (reviewed in [49])*.* The *SA INDUCTION-DEFICIENT2 (SID2)* locus, also known as *EDS16,* encodes an enzyme required for SA synthesis [50–52]. Both *eds1*-2 and *eds16-*2 behaved similar to the parental lines and flowered early at 27 °C (Figure 4A and Table S1), indicating that the SA pathway does not mediate thermal induction of flowering.

### Molecular Fingerprints of Temperature- and Light-Mediated Floral Induction

Since genetic analysis indicated that temperature acts independently of the photoperiod pathway, we went on to study molecular changes caused by thermal induction. To assess whether the genomic responses to thermal and photoperiodic induction are different, we first grew plants in 16 °C short days for 5 wk, after which they were still vegetative. We then changed either the photoperiod or the temperature; one set of plants was transferred to 16 °C long days, while the other set was transferred to 25 °C short days. Using a similar design as described [53], we analyzed changes in the transcriptome of shoot apices with Affymetrix ATH1 arrays on days 2, 5, and 9 after the transfer.

**Figure 5:**
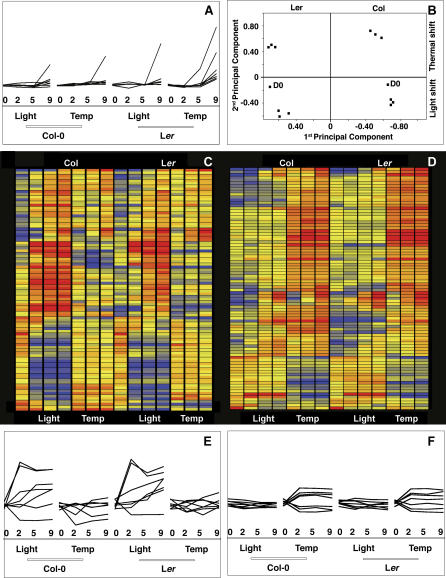
Genomic Responses at the Shoot Apex to Light or Temperature Treatment For (A), (E), and (F), the day of sample collection (0, 2, 5, and 9), type of shift (light-photoperiodic shift, temp-thermal shift) and the background (Col-0 and L*er*) are given in the *x*-axis. Log-normalized expression levels are plotted along the *y*-axis. The scale is the same for all three panels. (A) Response of floral marker genes *(AP1, FUL, AP3, PI, AG, SEP1–3)* to light and temperature shifts. (B) Principal component analysis. *x*-axis: first principal component explaining 39.5% of the variation, which appears to be mostly due to genetic differences between L*er* and Col (indicated above). *y*-axis: second principal component explaining 25% of the variation. The second component mostly distinguishes light versus temperature treatment (shown to the right). (C, D) Most genes that show alterations in expression levels (significantly different between day 0 and day 9 based on logit-T) appear to be specific to the type of induction (thermal or photoperiodic). Red indicates expression levels above average across all experiments; blue, levels below average. The left panel shows genes that are induced by light (top) or repressed by light (bottom), but largely unchanged in response to temperature. The right panel shows genes with the opposite behavior. (E) Examples of light specific changes in expression profiles *(CCA1, GI, COL2, SUMO3, AGL6, CRC,* and *TFL1).* (F) As examples of temperature specific changes in expression profiles, several genes encoding SR proteins and genes associated with the Gene Ontology term “RNA processing” are shown *(At2g24590, At5g46250, At1g55310, At1g09140, At1g51510* and *At2g27230).*

Several genes, including *SOC1, FRUITFUL (FUL), APETALA1 (AP1), CAULIFLOWER (CAL), LEAFY, SQUAMOSA PROMOTER BINDING PROTEIN LIKE 3 (SPL3),* and *SPL4*, are robust early markers for flower development [53]. On day 9, expression levels of these markers were substantially elevated, with several of them responding earlier and more strongly to thermal than to photoperiodic treatment (Figure 5A).

We employed several approaches to test how the genomic responses to thermal and photoperiodic induction differ. First, we used principal component analysis, which showed that transcriptomes of plants assayed at different time points in 25 °C short days were more similar to each other than those of plants in 16 °C long days, and that the environmental variable was more important than the temporal component (Figure 5B). Hierarchical clustering of conditions supported this finding (unpublished data). Because many genes responded to both treatments, we repeated this analysis with genes that were different on day 9 in both conditions compared to day 0. Even then the thermal and photoperiodic shift conditions clustered independently (unpublished data), confirming that the kinetics of induction of common targets is different.

### Light-Specific Genomic Responses

By comparing the transcriptome after either thermal or photoperiodic induction, we identified several genes whose profiles responded specifically to only one of the treatments (Figure 5C). We used the GeneMerge tool [54] to determine whether gene products involved in specific biological processes or molecular functions, as defined by their Gene Ontology annotations, preferentially responded to one of the treatments. Because early floral markers, such as *SPL3,* were already induced on day 2 in 25 °C short days, we focused on this timepoint.

In 16 °C long days, there was a significant enrichment of genes encoding zinc-binding proteins (*p* = 0.0067 after correcting for multiple testing), with two upregulated and four downregulated genes. All of them belong to B-box type zinc finger transcription factors. Several members of this family are clock-regulated [55], suggesting that this enrichment is due to an altered circadian profile in response to longer photoperiods (Figure 5D). This raised the question whether all the genes that are altered during light shift could simply be circadian-regulated genes. Therefore, we visually inspected the genes that show an altered profile during light shift for their behavior in a diurnal dataset [56]. More than three-quarters of the genes that had responded to photoperiod change by day 2 showed diurnal oscillations. This fraction was reduced to about two-thirds by day 9. In contrast, only about a quarter of the genes that responded to the thermal shift showed diurnal oscillations.

### Heat Shock and Thermal Induction of Flowering

A typical heat shock does not induce flowering [57]. In addition, our plants flowered early regardless of whether they were transferred at the adult stage to higher temperatures, or were grown from germination at elevated temperatures. In the latter case, plants should have become acclimated by the age that they are responsive to floral induction, and one would therefore not expect a heat shock effect on flowering. Nevertheless, we wanted to know whether heat shock–responsive genes were affected by our treatment.

An analysis of the microarray data showed that both the thermal and the photoperiodic shifts affected a small number of heat shock genes. However, none of the timepoints showed an enrichment for heat shock genes in thermally induced samples compared to photoperiod-induced samples. Among the genes highly induced in the thermal samples was *At3g12580,* one of the 14 *HSP70* genes encoded in the genome. Although *At3g12580* is stress responsive [58], it also has a complex developmentally regulated pattern of RNA expression [59]. Furthermore, it is well known that *HSP70* genes are involved in many different biological processes apart from heat shock responses [60].

Because heat shock genes typically respond rapidly and strongly to elevated temperatures, but return within hours to normal levels, our first microarray timepoint (48 h) might not have been appropriate for the analysis of heat shock genes. We therefore specifically compared the expression of several genes encoding heat shock proteins (HSPs) or heat shock transcription factors in samples shifted from 16 °C to 25 °C or 37 °C after 2 h. The selection of these genes was based on a previous detailed analysis of their response to heat shock [61]. As expected, several genes were strongly induced by 37 °C, while 25 °C had only minor effects (Figure S2), confirming that the thermal induction we find is not due to a typical heat shock response.

### Enrichment of RNA-Processing–Related Gene Products upon Thermal Induction

In 25 °C short days, we found a significant enrichment of genes encoding proteins involved in RNA processing (*p* = 0.007 after correcting for multiple testing; Figure 5E). This group included 11 factors associated with splicing or having RNA recognition motifs. Six genes encoding SR proteins, which are thought to control splice site selection and alternative splicing both in *Arabidopsis* as well as rice were upregulated [62,63]. Some of these factors have tissue-specific expression profiles and are expressed in the shoot apical meristem [59,62]. In addition, overexpression of *RSZ33* and *SR30* has pleiotropic effects that include variable alterations in flowering time [64,65]. RSZ33 interacts with RSZ21, another splicing factor [65], which was also upregulated.

The enrichment of RNA-processing related gene products responding to thermal induction suggests that temperature might affect RNA processing in *Arabidopsis.* This is particularly interesting, since there are several flowering time regulators with alternatively spliced transcripts [66]. Therefore, in a first step, we assessed *FCA, MAF2,* and *FLM,* genes known to have different splice forms. We detected different splice forms for all three genes. Using regular RT-PCR experiments, no obvious changes in the abundance of different *FCA* splice forms were detected (Figure S3), but more sensitive real-time RT-PCR experiments using splice-form–specific primers showed that there appears to be a subtle change in the relative abundance of the *FCA* splice forms, with a specific increase in the beta form during thermal shift (unpublished data). However, this form has no obvious function [67].

For *MAF2,* two splice forms of similar abundance were detected at 16 °C, before and after transfer to long days (Figure S3). In contrast, the larger splice form was more abundant in 25 °C short days. These differences in splicing patterns were consistent in three independent shift experiments (unpublished data).

For *FLM,* larger splice forms, albeit of minor abundance, appeared specifically after thermal shift, which seemed to be accompanied by a reduction in the levels of the major splice form (Figure S3). While it is tempting to speculate that this reduction could be a mechanism through which temperature overcomes the repressive effect of *FLM,* the function of the different splice forms is not yet known. Nevertheless, the observed changes in *FLM* are consistent with a role of *FLM* in this process. In addition, *flm* mutants flower earlier than *flc* mutants in 23 °C short days [11], suggesting that the repressive effects of *FLM* may be more important for the later flowering of plants grown in 23 °C compared to 25 °C or 27 °C short days. Conversely, the observed changes in *FCA* splice forms in response to elevated temperature may not be that important, since the *FCA* target *FLC* is still a potent repressor of flowering under these conditions. Further experiments are required to understand the specific effects of the different *FLM* and *MAF2* splice forms. Similarly, it remains to be tested whether the observed change in expression of SR protein genes directly affects these splicing patterns.

### Conclusions

We have shown that elevated temperature has a strong inductive effect on flowering, even in the absence of photoperiodic cues. Among wild accessions, there is tremendous variation in this response, which is partially attributable to the suppressive effect of *FLC* on flowering. Both mutant analysis and QTL mapping demonstrate that thermal induction has a genetic basis and acts in the same genetic cascade as *FLM.* Importantly, the genomic response to thermal induction differs from that of photoperiodic induction. Preliminary analyses suggest that altered splicing may be a component of thermal response in plants.

## Materials and Methods

### Plant work.

Seeds were obtained from the Nottingham *Arabidopsis* Stock Centre and from colleagues. The stock numbers of accessions and mutants used are given in Table S1. T-DNA insertion lines for *CO (co-9)* were obtained from Syngenta (Garlic-24-H04.b.1a.Lb3Fa; Basel, Switzerland). A SALK T-DNA line (Stock Centre number N641971) for *FLM* was isolated *(flm-3)* and verified to be an RNA-null allele (Min-Chul Kim and DW, unpublished data). Plants were cultivated in paired incubators (Percival Scientific, Perry, Iowa, United States) or growth rooms. Short days were 8 h of light/16 h of dark; long days were 16 h of light/8 h of dark. Ten to 12 plants per genotype were grown in a completely randomized design, in order to minimize environmental variation, and scored for their flowering time, which was measured by counting total leaf number (TLN, partitioned into Rosette [RLN] and Cauline [CLN] leaf number). Thermal induction was robust at 25 °C in incubators, but required 27 °C in the growth rooms. Therefore, induction experiments in the growth rooms were done at 27 °C. Growth chamber experiments were done at 25 °C, including microarray studies. There were no strong differences in the spectral quality between the chambers and the growth rooms, indicating that the observed differences could possibly be due to small temperature fluctuations in the chambers compared to the better ventilated growth rooms, which have only little variation (±0.1 °C).

### Expression studies.

Plants were grown for 5 wk at 16 °C in short days, and then transferred to either 16 °C long days or 25 °C short days. Twenty-five apices per genotype/condition/replicate were dissected and flash frozen on the day of the transfer (day 0) and days 2, 5, and 9 after transfer. RNA extracted from two independent biological replicate samples was hybridized to ATH1 arrays (Affymetrix, Santa Clara, California, United States) as previously described [53]. Data were normalized using the gcRMA algorithm (bioconductor.org), a modification of the robust multiarray analysis (RMA) algorithm [68], and visualized using Gene Spring 7 (Agilent Technologies, Palo Alto, California, United States). Pairwise comparisons were performed using logit-transformed probe-level testing [69]. Microarray data has been deposited with the ArrayExpress database (Supplemental Information). In addition to the RNA samples used for the microarray experiment, samples collected independently using a similar design were used for RT-PCR analysis to verify variations in splicing patterns. The primers used for the analysis of splicing patterns are given in Table S3. For circadian profiling of *FT,* aerial parts of 2-wk-old seedlings were collected over a 24-h time period in biological replicates. For the analysis of heat shock proteins, plants grown in 16 °C long days were shifted to 25 °C or 37 °C, and leaf material was collected at 30-min intervals for 2 h after the shift.

### Statistical analysis and QTL mapping.

Statistical analysis was done using the JMP package (SAS Institute, Cary North Carolina, United States), the statistical package R [70] (http://www.r-project.org), and Microsoft Excel. The NdC population has been described [24]. Twelve plants per RIL were grown at 27 °C short days in growth rooms in a completely randomized design. Broad-sense heritability *(H^2^)* was calculated as between-line variance *(V_G_)* divided by total variance. The total variance was partitioned into between-line variance and the residuals in a one-way ANOVA model using the genotype as a single factor of random effect and the TLN as the response. The estimated heritability was 0.62. QTL mapping was performed using R-qtl [71]. Likelihood of odds thresholds were determined using 1,000 permutations. Sensitivity to temperature was assessed through regression of the sample mean on the environmental mean as previously described [11]. Temperature sensitivity was calculated for each of the RILs, mutants and the accessions. The sensitivity measures obtained for the RILs were then used in QTL mapping as a phenotype to identify a QTL for thermosensitivity. The phenotypic measurements and the genotypic data used [24] for generating the QTL map are available in CSV format (NdC.csv).

## Supporting Information

Dataset S1 Dataset Used for Generating QTL Maps (21 KB CSV)

Figure S1 *FT* Expression Profiles in L*er* Plants Measured by qRT-PCR Three-wk-old plants grown at 23 °C and 27 °C in short days were analyzed. Dawn was at 9 AM, and dusk at 5 PM. *FT* levels are normalized to *FT* expression at 9 AM in 23 °C short-day samples, using tubulin RNA levels to standardize efficiency of qRT-PCR. Average results from two technical and two biological replicates are shown.
(273 KB TIF)

Figure S2 Response of Several Genes Encoding HSPs or Heat Shock Transcription Factors in Apices of Plants Transferred from 16 °C to Either 37 °C or 25 °C, as Measured by Real-Time RT-PCR Measurements in L*er* are shown on top, in Col on the bottom. 37 °C curves are indicated by asterisks. See [61] for primers.
(56 MB PDF)

Figure S3 Splicing Patterns Observed at the Shoot Apices of L*er* Plants in Response to Thermal and Light Induction Numbers on top refer to days after shift. See Table S3 for oligonucleotide primers used.
(1.9 MB TIF)

Table S1 Flowering Times of Accessions and Mutants in Short Days at 23 °C and 27 °C Bck*-genetic background. Sensitivity is the slope of the regression of total leaf number at 23 °C and 27 °C on the mean TLN at 23 °C and 27 °C.
(241 KB DOC)

Table S2 Relative Rankings of Accessions in 23 °C and 27 °C Short Days Earliest accession is ranked 1. Accessions with missing data were omitted in the analysis. Table is sorted according to the rankings at 27 °C.
(101 KB DOC)

Table S3 Primers Used for Analyzing the Variation in Splicing Patterns F, forward primer; R, reverse primer. *FLM/MAF1* and *MAF2* forward primers contain added restriction enzyme sites given by small letters. For *FCAγ* and *FCAδ,* the same reverse primers were used.
(33 KB DOC)

### Accession Numbers

The ArrayExpress (http://www.ebi.ac.uk/arrayexpress) accession number for the temperature/photoperiodic shift microarray experiment is E-MEXP-728.

## Abbreviations

ABA: abscisic acid
Col: Columbia strain
GA: gibberellin
GI: gigantea
HSP: heat shock protein
L*er*: Landsberg *erecta* strain
NdC: Niederzenz-1/Col
QTL: quantitative trait locus
RIL: recombinant inbred line
SA: salicylic acid
TLN: total leaf number
