# Supplementary material for: Potent Induction of Arabidopsis thaliana Flowering by Elevated Growth Temperature
Source: PLoS Genet. 2006 Jul 7;2(7):e106. doi: 10.1371/journal.pgen.0020106 (PMC1487179; doi:10.1371/journal.pgen.0020106)
Supplement: Supplementary file 1 [file pgen.0020106.sg002.pdf]

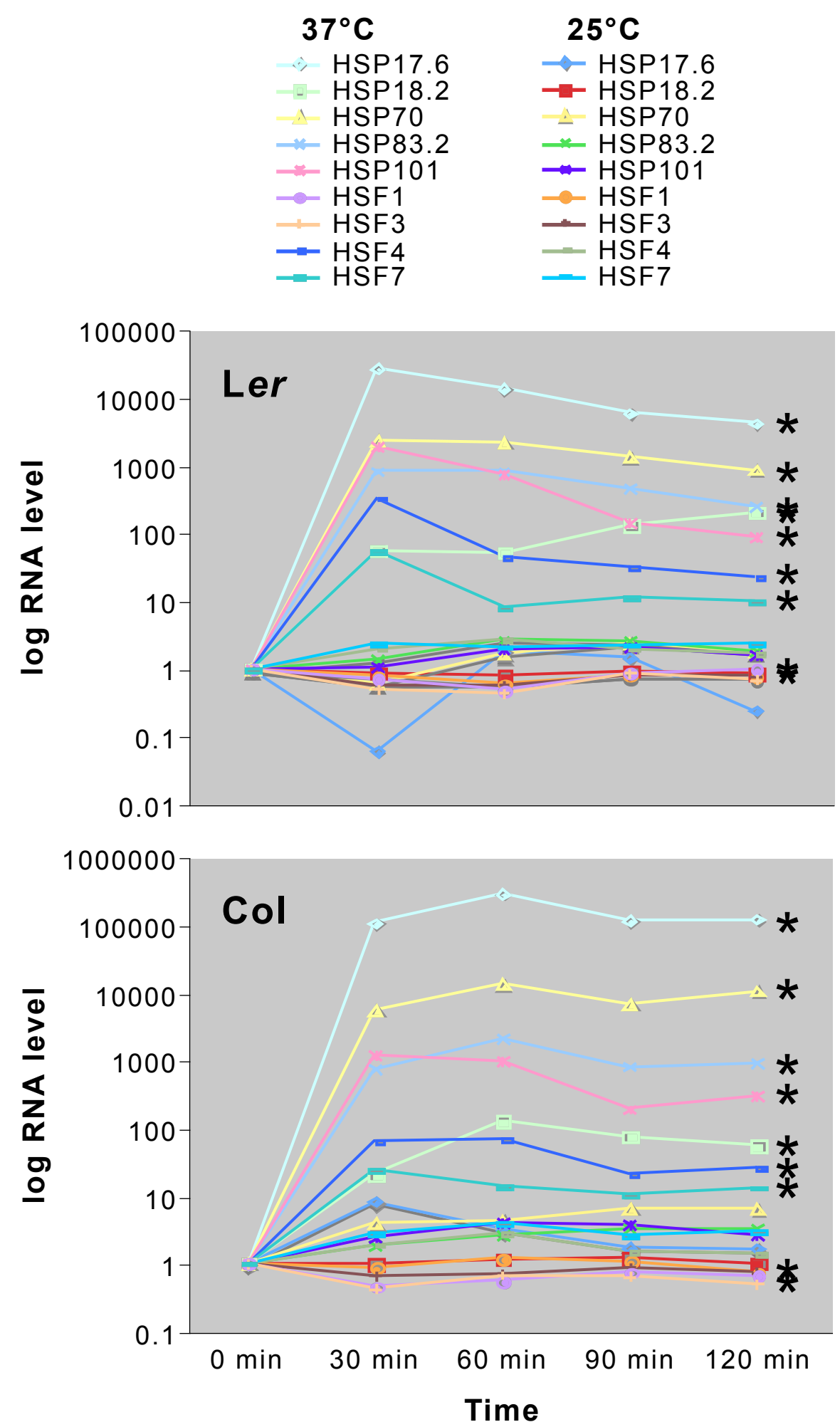
37°C
HSP17.6
HSP18.2
HSP70
HSP83.2
HSP101
HSF1
HSF3
HSF4
HSF7
25°C
HSP17.6
HSP18.2
HSP70
HSP83.2
HSP101
HSF1
HSF3
HSF4
HSF7
Ler
log RNA level
100000
10000
1000
100
10
1
0.1
0.01
Col
log RNA level
1000000
100000
10000
1000
100
10
1
0.1
0 min
30 min
60 min
90 min
120 min
Time
