# Supplementary material for: Potent Induction of Arabidopsis thaliana Flowering by Elevated Growth Temperature
Source: PLoS Genet. 2006 Jul 7;2(7):e106. doi: 10.1371/journal.pgen.0020106 (PMC1487179; doi:10.1371/journal.pgen.0020106)
Supplement: Supplementary file 2 [file pgen.0020106.st001.doc]

**Supplementary Table 1.** Flowering times of accessions and mutants in short days at 23°C and 27°C.

Bck*-Genetic background. Sensitivity is the slope of the regression of total leaf number at 23°C and 27°C on the mean total leaf number at 23°C and 27°C.

| **S.No** | **Genotypes** | **Stock Number** | **FRI/FLC** | **RLN23SD** | **CLN23SD** | **TLN23SD** | **RLN27SD** | **CLN27SD** | **TLN27SD** | **Sensitivity** |
| --- | --- | --- | --- | --- | --- | --- | --- | --- | --- | --- |
|  |  |  |  |  |  | **Accessions** |  |  |  |  |
| 1 | Ang-1 | N951 | Del | 49.63 | 9.42 | 59.05 | 37.43 | 7.86 | 45.29 | 0.85 |
| 2 | Bla-5 | N6620 |  | 55.00 | ND | 55.00 | ND | ND | ND | ND |
| 3 | Bs-5 | N1001 | Del | 34.09 | 7.36 | 41.45 | 17.43 | 4.43 | 21.86 | 1.21 |
| 4 | Bsch-2 | N1005 | Del | 31.25 | 8.92 | 40.17 | 13.00 | 3.88 | 16.88 | 1.44 |
| 5 | Bur-0 | N1029 |  | 56.57 | 7.00 | 63.57 | ND | ND | ND | ND |
| 6 | C24 | Lab Stock |  | 41.33 | 13.33 | 54.67 | 33.19 | 11.08 | 44.27 | 0.64 |
| 7 | Cen-0 | N1065 | Del | 62.19 | 15.95 | 78.14 | 53.25 | 11.75 | 65.00 | 0.81 |
| 8 | Chi-0 | N1073 |  | 49.33 | 7.67 | 57.00 | 43.00 | 8.13 | 51.13 | 0.36 |
| 9 | Coimbra-1 | N1085 |  | 39.00 | 12.08 | 51.08 | 32.50 | 10.25 | 42.75 | 0.52 |
| 10 | Col-0 | Lab Stock | Del | 49.10 | 9.93 | 59.03 | 25.46 | 4.44 | 29.90 | 1.81 |
| 11 | Cvi-0 | N1097 | Del | 19.20 | 4.20 | 23.40 | 19.89 | 3.89 | 23.78 | -0.02 |
| 12 | Dra-1 | N1119 | Del | 30.50 | 5.58 | 36.08 | 12.38 | 3.75 | 16.13 | 1.24 |
| 13 | Ei-6 | N1131 |  | ND | ND | 24.89 | ND | ND | 15.28 | 0.60 |
| 14 | Est-1 | N1151 | Del | 33.53 | 11.37 | 44.89 | 11.97 | 3.90 | 15.87 | 1.80 |
| 15 | Fr-2 | N1169 | Del | 20.90 | 6.40 | 27.30 | 11.50 | 4.25 | 15.75 | 0.72 |
| 16 | Ga-0 | N1181 | Del | 52.35 | 8.59 | 60.94 | 50.06 | 8.06 | 58.11 | 0.18 |
| 17 | Ge-1 | N1189 |  | 70.22 | 9.89 | 80.11 | DNF | DNF | DNF | ND |
| 18 | Go-2 | N1197 | Del | 44.92 | 12.83 | 57.75 | 30.38 | 8.13 | 38.50 | 1.19 |
| 19 | GOT1 | N22277 |  | 59.00 | 10.50 | 69.50 | DNF | DNF | DNF | ND |
| 20 | Gre-0 | N1211 |  | DNF | DNF | DNF | DNF | DNF | DNF | ND |
| 21 | Hau-0 | N1221 | Del | 37.25 | 9.00 | 46.25 | 23.75 | 6.25 | 30.00 | 1.01 |
| 22 | HR15 | N22214 | Del | 43.25 | 12.42 | 55.67 | 31.29 | 10.71 | 42.00 | 0.85 |
| 23 | In-0 | N1239 |  | 51.71 | 10.57 | 62.29 | 56.50 | 9.33 | 65.83 | -0.22 |
| 24 | Khodja-Obi | N6178 | Del | 50.08 | 5.58 | 55.67 | 21.00 | 1.75 | 22.75 | 2.04 |
| 25 | Kil-0 | N1271 | Del | 32.42 | 10.58 | 43.00 | 20.00 | 5.50 | 25.50 | 1.08 |
| 26 | Kin-0 | N1273 | Del | 28.60 | 6.70 | 35.30 | 16.62 | 4.48 | 21.10 | 0.88 |
| 27 | Kn-0 | N1287 | Del | 39.27 | 9.64 | 48.91 | 25.25 | 6.38 | 31.63 | 1.07 |
| 28 | Kondara | N6175 | Del | 51.42 | 5.83 | 57.25 | 24.00 | 1.13 | 25.13 | 1.99 |
| 29 | Kyoto-3 | JW139 |  | 49.73 | 12.09 | 61.82 | 38.29 | 10.00 | 48.29 | 0.84 |
| 30 | KZ10 | N22442 |  | 40.27 | 7.82 | 48.09 | 18.38 | 4.25 | 22.63 | 1.58 |
| 31 | Ler | Lab Stock | Del | 27.65 | 10.48 | 38.13 | 9.22 | 3.51 | 12.73 | 1.57 |
| 32 | Lip-0 | N1337 | Del | 28.09 | 9.27 | 38.18 | 17.63 | 6.63 | 24.25 | 0.86 |
| 33 | Mh-1 | N1369 |  | 40.17 | 12.83 | 53.00 | 20.38 | 5.38 | 25.75 | 1.69 |
| 34 | Mir-0 | N1379 |  | 62.00 | 2.00 | 64.00 | DNF | DNF | DNF | ND |
| 35 | Ms-0 | N1377 |  | 56.00 | 11.00 | 67.00 | 61.60 | 8.80 | 70.40 | -0.21 |
| 36 | Mt-0 | N1381 | Del | 36.38 | 8.75 | 45.13 | 15.00 | 3.73 | 18.73 | 1.64 |
| 37 | Nd-1 | N1680 | Del | 32.56 | 11.11 | 43.67 | 26.64 | 8.23 | 34.86 | 0.55 |
| 38 | Newzealand | Skotnicki Lab |  | 36.17 | 9.08 | 45.25 | 17.00 | 3.75 | 20.75 | 1.52 |
| 39 | Nok-0 | N1399 |  | 60.33 | 8.56 | 68.89 | 43.86 | 6.57 | 50.43 | 1.14 |
| 40 | Pa-2 | N1441 |  | 80.00 | 13.63 | 93.63 | 65.50 | 12.25 | 77.75 | 0.98 |
| 41 | Per-2 | N1449 |  | 59.00 | 5.33 | 64.33 | 55.00 | 7.60 | 62.60 | 0.11 |
| 42 | Pi-0 | N1455 | Del | 34.58 | 8.58 | 43.17 | 25.50 | 6.38 | 31.88 | 0.70 |
| 43 | Pog-0 | N1477 | Del | 57.67 | 10.33 | 68.00 | 56.00 | 7.00 | 63.00 | 0.31 |
| 44 | REN1 | N22253 |  | 44.58 | 10.58 | 55.17 | 39.38 | 6.50 | 45.88 | 0.58 |
| 45 | Ri-0 | N1493 |  | 52.42 | 10.75 | 63.17 | 58.17 | 8.67 | 66.83 | -0.23 |
| 46 | Sf-2 | N1517 |  | 48.11 | 8.67 | 56.78 | 44.50 | 8.25 | 52.75 | 0.25 |
| 47 | Shakdara | N6180 | Del | 19.00 | 5.80 | 24.80 | 8.09 | 2.50 | 10.59 | 0.88 |
| 48 | Ta-0 | N1549 |  | 48.83 | 9.83 | 58.67 | 35.71 | 7.43 | 43.14 | 0.96 |
| 49 | Tsu-1 | N6926 | Del | 36.86 | 10.55 | 47.41 | 20.87 | 6.53 | 27.40 | 1.24 |
| 50 | Uk-1 | N1575 | Del | 70.86 | 13.71 | 84.57 | 51.00 | 10.40 | 61.40 | 1.44 |
| 51 | Uk-3 | N1577 | Del | 38.70 | 6.60 | 45.30 | 37.50 | 6.14 | 43.64 | 0.10 |
| 52 | Van-0 | CS6884 | Del | 60.40 | 12.40 | 72.80 | 27.38 | 10.25 | 37.63 | 2.18 |
| 53 | Wc-1 | N1589 | Del | 26.58 | 10.50 | 37.08 | 23.50 | 9.25 | 32.75 | 0.27 |
| 54 | Wil-1 | N1595 | Del | 33.36 | 10.00 | 43.36 | 22.00 | 7.00 | 29.00 | 0.89 |
|  | **Average** |  |  | **44.47** | **9.44** | **53.20** | **30.81** | **6.73** | **37.07** | **0.91** |
|  | **SD** |  |  | **13.70** | **2.72** | **15.00** | **15.62** | **2.72** | **17.75** | **0.62** |
|  |  |  |  |  |  | **Mutants** |  |  |  |  |
| **S.No** | **Genotypes** |  | **Bck*** | **RLN23SD** | **CLN23SD** | **TLN23SD** | **RLN27SD** | **CLN27SD** | **TLN27SD** | **Sensitivity** |
| 1 | Col-0 | Lab Stock | Col | 49.10 | 9.93 | 59.03 | 25.46 | 4.44 | 29.90 | 1.89 |
| 2 | co-9 | Syngenta | Col | 45.08 | 9.92 | 55.00 | 23.13 | 3.63 | 26.75 | 1.83 |
| 3 | cry-2-1 | N3732 | Col | 50.32 | 10.23 | 60.55 | 31.00 | 5.63 | 36.63 | 1.55 |
| 4 | eds16-1 | Dangl_Lab | Col | 59.86 | 9.14 | 69.00 | 30.14 | 4.57 | 34.71 | 2.22 |
| 5 | fcaflc | Amasino_Lab | Col | 42.25 | 8.75 | 51.00 | 16.14 | 2.86 | 19.00 | 2.07 |
| 6 | fkf1 | Lab Stock | Col | 48.13 | 9.33 | 57.46 | 40.75 | 8.13 | 48.88 | 0.56 |
| 7 | flc-3 | Lab Stock | Col | 45.95 | 9.03 | 54.97 | 16.29 | 3.59 | 19.88 | 2.28 |
| 8 | fld-1 | Amasino_Lab | Col | 55.80 | 9.30 | 65.10 | 57.00 | 7.00 | 64.00 | 0.07 |
| 9 | fld-1 flc-3 | Amasino_Lab | Col | 48.22 | 11.87 | 60.09 | 20.88 | 3.63 | 24.50 | 2.31 |
| 10 | fpa-1 flc-3 | Amasino_Lab | Col | 48.11 | 11.33 | 59.44 | 17.38 | 2.38 | 19.75 | 2.57 |
| 11 | ft-1 | Lab Stock | Col | 48.17 | 12.00 | 60.17 | 49.29 | 9.86 | 59.14 | 0.07 |
| 12 | gi-2 | Lab Stock | Col | 52.33 | 10.25 | 62.58 | 49.43 | 8.71 | 58.14 | 0.29 |
| 13 | ld | Amasino_Lab | Col | DNF | DNF | DNF | DNF | DNF | DNF | ND |
| 14 | ldflc | Amasino_Lab | Col | 47.67 | 11.67 | 59.33 | 15.75 | 3.00 | 18.75 | 2.63 |
| 15 | flm-3 | SALK line | Col | 30.50 | 6.33 | 36.83 | 17.75 | 2.88 | 20.63 | 1.05 |
| 16 | Ler | Lab Stock | Ler | 27.65 | 10.48 | 38.13 | 9.22 | 3.51 | 12.73 | 1.65 |
| 17 | 35S::FCA | Dean_Lab | Ler | 21.83 | 9.00 | 30.83 | 9.25 | 4.75 | 14.00 | 1.09 |
| 18 | cca1-100 | Mizoguchi_Lab | Ler | 23.08 | 9.67 | 32.75 | 10.50 | 4.00 | 14.50 | 1.18 |
| 19 | co-1 | N3122 | Ler | 25.94 | 10.00 | 35.94 | 16.91 | 6.91 | 23.82 | 0.79 |
| 20 | co-2 | Lab Stock | Ler | 26.03 | 9.79 | 35.83 | 12.61 | 5.39 | 18.00 | 1.16 |
| 21 | co-8 | Coupland_Lab | Ler | ND | ND | ND | 14.13 | 6.00 | 20.13 | ND |
| 22 | eds1-2 | Dangl_Lab | Ler | ND | ND | ND | 8.20 | 4.55 | 12.75 | ND |
| 23 | efs | Lab Stock | Ler | 15.00 | 3.83 | 18.83 | 9.00 | 4.00 | 13.00 | 0.38 |
| 24 | fca-1 | N52 | Ler | 42.81 | 9.29 | 52.10 | 49.67 | 7.33 | 57.00 | -0.32 |
| 25 | fca-2 | N168 | Ler | 31.05 | 10.82 | 41.86 | 34.29 | 8.86 | 43.14 | -0.08 |
| 26 | fd-1 | Lab Stock | Ler | 22.84 | 8.19 | 31.03 | 18.00 | 7.13 | 25.13 | 0.38 |
| 27 | fe-1 | N54 | Ler | 26.04 | 10.82 | 37.21 | 17.14 | 8.39 | 25.54 | 0.76 |
| 28 | fha-1 | N108 | Ler | 29.74 | 8.82 | 38.56 | 13.13 | 4.13 | 17.25 | 1.38 |
| 29 | fha-2 | N186 | Ler | 22.91 | 10.27 | 33.18 | 11.00 | 4.71 | 15.71 | 1.13 |
| 30 | fha-3 | N187 | Ler | 23.82 | 9.89 | 33.71 | 10.65 | 4.46 | 15.12 | 1.21 |
| 31 | fpa-1 | N109 | Ler | 36.45 | 11.52 | 47.97 | 35.00 | 11.33 | 46.33 | 0.11 |
| 32 | ft-1 | Lab Stock | Ler | 27.84 | 11.40 | 39.24 | 18.00 | 8.67 | 26.67 | 0.81 |
| 33 | ft-2 | Lab Stock | Ler | 26.28 | 11.22 | 37.50 | 21.00 | 9.50 | 30.50 | 0.45 |
| 34 | ft-3 | Lab Stock | Ler | 28.80 | 11.10 | 39.90 | 24.50 | 9.13 | 33.63 | 0.41 |
| 35 | fve-1 | N107 | Ler | 47.00 | 10.06 | 57.06 | 52.06 | 8.81 | 60.88 | -0.25 |
| 36 | fwa-1 | N106 | Ler | 27.00 | 12.62 | 39.62 | 19.75 | 10.75 | 30.50 | 0.59 |
| 37 | fwa-2 | Lab Stock | Ler | 23.11 | 10.00 | 33.11 | 15.64 | 8.85 | 24.38 | 0.57 |
| 38 | fy-1 | N57 | Ler | 32.80 | 8.56 | 41.36 | 28.27 | 6.13 | 34.40 | 0.45 |
| 39 | ga1-3 | Lab Stock | Ler | DNF | DNF | DNF | DNF | DNF | DNF | ND |
| 40 | gai | Lab Stock | Ler | 30.60 | 5.00 | 35.60 | 22.90 | 3.20 | 26.10 | 0.62 |
| 41 | gi-3 | Lab Stock | Ler | ND | ND | ND | 10.39 | 3.88 | 13.88 | ND |
| 42 | gi-6 | N183 | Ler | 21.97 | 8.64 | 30.64 | 11.88 | 4.13 | 16.00 | 0.95 |
| 43 | hy4 | N70 | Ler | 44.13 | 11.06 | 55.19 | 10.33 | 4.50 | 14.83 | 2.62 |
| 44 | lhy-12 | Mizoguchi_Lab | Ler | 18.83 | 6.17 | 25.00 | 9.13 | 3.50 | 12.63 | 0.80 |
| 45 | lhy-12 cca1-1 | Mizoguchi_Lab | Ler | 6.90 | 2.40 | 9.30 | 6.25 | 2.25 | 8.50 | 0.05 |
| 46 | lhy-12 cca1-10 | Mizoguchi_Lab | Ler | 7.70 | 2.30 | 10.00 | 6.88 | 1.88 | 8.75 | 0.08 |
| 47 | soc1 | Coupland_Lab | Ler | 35.13 | 14.29 | 49.33 | 18.13 | 8.50 | 26.63 | 1.47 |
| 48 | soc1 ft-7 | Coupland_Lab | Ler | 34.40 | 12.35 | 46.52 | 25.86 | 14.43 | 40.29 | 0.40 |
| 49 | flm-1 | Lab Stock | Ws | 12.00 | 4.88 | 16.88 | 7.80 | 3.90 | 11.70 | 0.34 |
| 50 | flm-2 | Lab Stock | Ws | 10.67 | 4.67 | 15.33 | 7.90 | 3.40 | 11.30 | 0.26 |
