# Supplementary material for: Potent Induction of Arabidopsis thaliana Flowering by Elevated Growth Temperature
Source: PLoS Genet. 2006 Jul 7;2(7):e106. doi: 10.1371/journal.pgen.0020106 (PMC1487179; doi:10.1371/journal.pgen.0020106)
Supplement: Supplementary file 3 [file pgen.0020106.st002.doc]

**Supplementary Table 2.** Relative rankings of accessions in 23°C and 27°C short days.

Earliest accession is ranked 1. Accessions with missing data were omitted in the analysis. Table is sorted according to the rankings at 27°C.

| **S.No** | **Genotypes** | **TLN23SD** | **TLN27SD** | **23°C Rank** | **27°C Rank** |
| --- | --- | --- | --- | --- | --- |
| 1 | Shakdara | 24.80 | 10.59 | 2 | 1 |
| 2 | Ler | 38.13 | 12.73 | 7 | 2 |
| 3 | Fr-2 | 27.30 | 15.75 | 3 | 3 |
| 4 | Est-1 | 44.89 | 15.87 | 15 | 4 |
| 5 | Dra-1 | 36.08 | 16.13 | 5 | 5 |
| 6 | Bsch-2 | 40.17 | 16.88 | 9 | 6 |
| 7 | Mt-0 | 45.13 | 18.73 | 16 | 7 |
| 8 | New Zealand | 45.25 | 20.75 | 17 | 8 |
| 9 | Kin-0 | 35.30 | 21.10 | 4 | 9 |
| 10 | Bs-5 | 41.45 | 21.86 | 10 | 10 |
| 11 | KZ10 | 48.09 | 22.63 | 21 | 11 |
| 12 | Khodja-Obi | 55.67 | 22.75 | 28 | 12 |
| 13 | Cvi-0 | 23.40 | 23.78 | 1 | 13 |
| 14 | Lip-0 | 38.18 | 24.25 | 8 | 14 |
| 15 | Kondara | 57.25 | 25.13 | 31 | 15 |
| 16 | Kil-0 | 43.00 | 25.50 | 11 | 16 |
| 17 | Mh-1 | 53.00 | 25.75 | 24 | 17 |
| 18 | Tsu-1 | 47.41 | 27.40 | 20 | 18 |
| 19 | Wil-1 | 43.36 | 29.00 | 13 | 19 |
| 20 | Col-0 | 59.03 | 29.90 | 34 | 20 |
| 21 | Hau-0 | 46.25 | 30.00 | 19 | 21 |
| 22 | Kn-0 | 48.91 | 31.63 | 22 | 22 |
| 23 | Pi-0 | 43.17 | 31.88 | 12 | 23 |
| 24 | Wc-1 | 37.08 | 32.75 | 6 | 24 |
| 25 | Nd-1 | 43.67 | 34.86 | 14 | 25 |
| 26 | Van-0 | 72.80 | 37.63 | 44 | 26 |
| 27 | Go-2 | 57.75 | 38.50 | 32 | 27 |
| 28 | HR15 | 55.67 | 42.00 | 27 | 28 |
| 29 | Coimbra-1 | 51.08 | 42.75 | 23 | 29 |
| 30 | Ta-0 | 58.67 | 43.14 | 33 | 30 |
| 31 | Uk-3 | 45.30 | 43.64 | 18 | 31 |
| 32 | C24 | 54.67 | 44.27 | 25 | 32 |
| 33 | Ang-1 | 59.05 | 45.29 | 35 | 33 |
| 34 | REN1 | 55.17 | 45.88 | 26 | 34 |
| 35 | Kyoto-3 | 61.82 | 48.29 | 37 | 35 |
| 36 | Nok-0 | 68.89 | 50.43 | 43 | 36 |
| 37 | Chi-0 | 57.00 | 51.13 | 30 | 37 |
| 38 | Sf-2 | 56.78 | 52.75 | 29 | 38 |
| 39 | Ga-0 | 60.94 | 58.11 | 36 | 39 |
| 40 | Uk-1 | 84.57 | 61.40 | 46 | 40 |
| 41 | Per-2 | 64.33 | 62.60 | 40 | 41 |
| 42 | Pog-0 | 68.00 | 63.00 | 42 | 42 |
| 43 | Cen-0 | 78.14 | 65.00 | 45 | 43 |
| 44 | In-0 | 62.29 | 65.83 | 38 | 44 |
| 45 | Ri-0 | 63.17 | 66.83 | 39 | 45 |
| 46 | Ms-0 | 67.00 | 70.40 | 41 | 46 |
| 47 | Pa-2 | 93.63 | 77.75 | 47 | 47 |
