# Supplementary material for: Potent Induction of Arabidopsis thaliana Flowering by Elevated Growth Temperature
Source: PLoS Genet. 2006 Jul 7;2(7):e106. doi: 10.1371/journal.pgen.0020106 (PMC1487179; doi:10.1371/journal.pgen.0020106)
Supplement: Supplementary file 4 [file pgen.0020106.st003.doc]

**Supplementary Table 3.** Primers used for analyzing the variation in splicing patterns.

*F-forward primer, R-reverse primer. FLM/MAF1 and MAF2 forward primers contain added restriction enzyme sites given by small letters. For FCAγ and FCA same reverse primers were used.*

| **S.No** | **Primer** | **Gene** | **Sequence (5’ to 3’)** |
| --- | --- | --- | --- |
| 1 | G-4084 | *FLM/MAF1_F* | cggatccccATGGGAAGAAGAAAAATCGAG |
| 2 | G-1978 | *FLM/MAF1_R* | TAATTGAGCAGCGGGAGAGT |
| 3. | G-4087 | *MAF2_F* | cggatccccATGGGTAGAAAAAAAGTCGAG |
| 4 | G-4089 | *MAF2_R* | GTCCCGGGCTTGAGCAGCGGAAGAGTCTCC |
| 5 | G-8005 | *FCAγ_F* | CAATTTCTCAGCTGCAGTAAGTC |
| 6 | G-1807 | *FCAγ_R FCAδ_R* | TGAAGAATAAAACGGCTGCTGTA |
| 7 | G-8006 | *FCAβ_F* | GAAGAAGAAATCCGTCCCTATTTCG |
| 8 | G-8009 | *FCAβ_R* | GCGAAATCTATTTGCCCAAC |
| 9 | G-1806 | *FCAδ_F* | CAATTTCTCAGCTGCAGTAAGTC |
| 10 | N-78 | *TUB_F* | GAGCCTTACAACGCTACTCTGTCTGTC |
| 11 | N-79 | *TUB_R* | ACACCAGACATAGTAGCAGAAATCAAG |
